# Probiotics and Maternal Mental Health: A Randomised Controlled Trial among Pregnant Women with Obesity

**DOI:** 10.1038/s41598-020-58129-w

**Published:** 2020-01-28

**Authors:** Julia P. Dawe, Lesley M. E. McCowan, Jess Wilson, Karaponi A. M. Okesene-Gafa, Anna S. Serlachius

**Affiliations:** 1 Department of Psychological Medicine, Faculty of Medical and Health Sciences, The University of Auckland, 1142 Auckland, New Zealand; 2 Department of Obstetrics and Gynaecology, Faculty of Medical and Health Sciences, The University of Auckland, 1142 Auckland, New Zealand

**Keywords:** Nutritional supplements, Weight management, Outcomes research

## Abstract

Poor maternal mental health has been associated with a myriad of pregnancy and child health complications. Obesity in pregnancy is known to increase one’s risk of experiencing poor maternal mental health and associated physical and mental health complications. Probiotics may represent a novel approach to intervene in poor mental health and obesity. We conducted this pre-specified secondary analysis of the Healthy Mums and Babies (HUMBA) randomised controlled trial to investigate whether probiotics would improve maternal mental health outcomes up to 36 weeks of pregnancy. Two-hundred-and-thirty pregnant women with obesity (BMI ≥ 30.0 kg/m^2^) were recruited and randomised to receive probiotic (*Lactobacillus rhamnosus* GG and *Bifidobacterium lactis* BB12, minimum 6.5 × 10^9^ CFU) or placebo capsules. Depression, anxiety, and functional health and well-being were assessed at baseline (12^0^−17^6^ weeks’ gestation) and 36 weeks of pregnancy. Depression scores remained stable and did not differ between the probiotic (*M* = 7.18, *SD* = 3.80) and placebo groups (*M* = 6.76, *SD* = 4.65) at 36 weeks (*p*-values > 0.05). Anxiety and physical well-being scores worsened over time irrespective of group allocation, and mental well-being scores did not differ between the two groups at 36 weeks. Probiotics did not improve mental health outcomes in this multi-ethnic cohort of pregnant women with obesity.

## Introduction

The perinatal period is a time of significant transition, wherein physical, psychological, and social changes occur, and new challenges are encountered^1–4^. Thus, it is unsurprising that maternal mental health issues, such as depression and anxiety, are commonly experienced by women during this time. The experience of poor maternal mental health has been associated with a myriad of adverse pregnancy and birth outcomes such as preterm birth^3,5–7^ and low infant birthweight^3,6^, in addition to longer-term negative impacts regarding child development^8–11^, offspring mental health^8,12,13^, early mother-child interaction^14–17^, and ongoing mental health issues for the mother^17,18^. Despite significant consequences, maternal mental health symptomology continues to be under-recognised and under-treated^2,19^.

Pregnancy with obesity represents an additional significant and prevalent health condition that can increase the risk of pregnancy complications and contribute to negative maternal and child outcomes that persist beyond the perinatal period^20–22^. Evidence suggests that obesity during pregnancy may be a factor contributing to an increased risk of adverse maternal mental health outcomes, as women who enter pregnancy with obesity have been found to be more likely to experience perinatal depression and anxiety symptoms relative to women who enter pregnancy at a normal or healthy weight^23–25^.

A potentially novel approach to targeting both obesity and poor mental health is through the modification of the gut microbiota and thus the gut-brain axis, via consumption of probiotics. Dysregulation of the gut-brain axis and alterations in the gut microbiota (i.e. dysbiosis) have been implicated in both obesity and mental health disorders, and emerging clinical studies suggest probiotics may be an effective means by which to address both^26–29^. Building on a plethora of pre-clinical studies, a plausible biological mechanism that supports this approach relates to the gut microbiota’s potential ability to regulate neuro-inflammation. It has been hypothesised that probiotics may be able to restore microbial dysbiosis, improve gastrointestinal integrity, and subsequently reduce inflammation and normalise neuroendocrine activity and neurotransmission, thereby targeting both obesity and mental health disorders via a shared mechanistic pathway^26,29–31^. Probiotics are considered safe during pregnancy, and are a simple, easy, and cost-effective intervention, that have previously demonstrated positive metabolic outcomes in randomised controlled trials with pregnant women^32,33^.

Research regarding mental health outcomes of pregnant women who consume probiotics is almost entirely absent from the literature, despite the growing body of studies exploring probiotics and mental health among non-pregnant populations, and studies exploring probiotics and other outcomes (e.g. metabolic) in pregnant populations. One study which has explored mental health outcomes in pregnant women with a history of asthma, eczema, or hayfever, demonstrated that those allocated to receive the probiotic *Lactobacillus rhamnosus* HN001 during pregnancy, had significantly lower anxiety and depression scores in the postnatal period compared to those who received a placebo^34^. There remains a clear need to further investigate this relationship between probiotics and maternal mental health, and additionally to investigate this relationship in the context of pregnant women with obesity.

No randomised controlled trials have investigated the influence of probiotics on mental health outcomes among pregnant women with obesity. To address this research gap and contribute to the growing body of literature on probiotics and mental health, the current study was conducted to investigate the influence of *Lactobacillus rhamnosus* GG and *Bifidobacterium lactis* BB12 on depression, anxiety, and functional health and well-being, among a multi-ethnic sample of pregnant women with obesity residing in the Counties Manukau Health (CMH) region in South Auckland, New Zealand. The CMH region is characterised by high levels of pregnancy with obesity and socioeconomic deprivation, and has a high percentage of vulnerable ethnic minority residents^35^, thus making it a pertinent population for mental health research and intervention. We hypothesised that women allocated to the probiotic capsule intervention would have improvements in depression, anxiety, and functional health and well-being scores from baseline to 36 weeks of pregnancy, in comparison to those in the placebo group. We also hypothesised that women allocated to the probiotic intervention would demonstrate lower depression and anxiety scores and higher functional health and well-being scores in comparison to the those in the placebo group at 36 weeks of pregnancy.

## Methods

### Design

This study was a pre-specified secondary analysis of the Healthy Mums and Babies (HUMBA) trial. The HUMBA trial was a single-centre two-by-two factorial randomised controlled demonstration trial (parallel groups), designed to investigate the influence of probiotic versus placebo capsules (double-blind), and dietary intervention versus routine dietary advice (no blinding) on several outcome measures among a sample of pregnant women with obesity and their offspring in the CMH region. While the primary outcomes of interest of the HUMBA trial were excessive gestational weight gain and infant birth weight, this secondary analysis pertains to antenatal mental health outcomes of the study cohort and focusses on the effects of the probiotic intervention. Detailed information pertaining to the overall HUMBA trial has been published elsewhere^36,37^. The HUMBA trial was designed in accordance with the CONSORT guidelines^38^.

### Trial registration and ethical approval

The HUMBA demonstration trial was registered with ANZCTR (ACTRN12615000400561). Ethical approval was granted by the Southern Health and Disabilities Ethics Committee (14/STH/205), and locality approval was established with Counties Manukau District Health Board (CMDHB). All participants provided written informed consent and the research was conducted in accordance with the Helsinki Declaration.

### Sample

Participants were recruited from within the CMH region between April 2015 and June 2017. Women were eligible and approached to participate in the study if they had a singleton pregnancy, were between 12°−17 weeks and 6 day’s gestation, had a BMI of ≥30.0 kg/m^2^, and were able to provide informed written consent. Participants were excluded if they had pre-existing diabetes or an HbA1c (average blood glucose) of ≥50 mmol/mol at time of recruitment, had known fetal congenital abnormalities, were already taking probiotic capsules or supplements containing probiotics, had a multiple pregnancy, had received bariatric surgery, were taking medications or had a medical condition that altered glucose metabolism, and/or had severe hyperemesis. Additionally, participants were excluded if they declined to participate or were unable to provide informed written consent.

**Figure 1:**
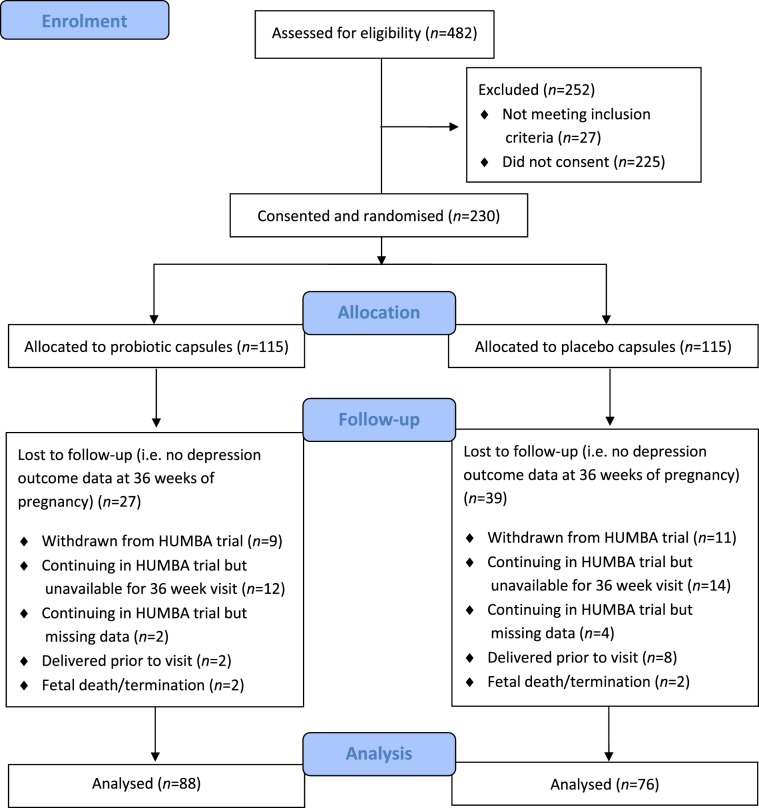
CONSORT diagram displaying flow of participant involvement in the study.

A total of 482 women were approached and assessed for eligibility to participate in the HUMBA trial. Two hundred and thirty met the eligibility criteria, consented to participate, and were randomised to receive either probiotic or placebo capsules. Of the 230 women recruited, primary outcome data pertaining to the current study (depression scores at 36 weeks of pregnancy) were obtained for 164 women, thus forming the analytic sample. See Fig. 1 for CONSORT diagram of participant flow.

Using G*Power 3.1^39^ it was calculated that the sample size of 164 participants was powered to detect a 2-point difference in mean Edinburgh Postnatal Depression Scale (EPDS)^40^ scores (Cohen’s *d* = 0.44), using an independent samples t-test with 80% power and an alpha of 0.05.

### Randomisation

Randomisation was performed using a web-based protocol (http://randomize.net/), with random block sizes that ranged from 4 to 8. Participants were stratified by BMI category (30.0–34.9 kg/m^2^ or ≥35.0 kg/m^2^), and then subsequently randomised to receive probiotic or placebo capsules using a 1:1 allocation ratio.

### Intervention

Participants randomised to the probiotic intervention received probiotic capsules containing *Lactobacillus rhamnosus* GG and *Bifidobacterium lactis* BB12 (Chr. Hansen A/S, Hoersholm, Denmark), at a minimum dose of 6.5 × 10^9^ colony forming units (CFU) per day. The probiotic formulation was based on that used in an earlier study which demonstrated reductions in gestational diabetes among pregnant women randomised to receive both dietary counselling and probiotic supplementation^32^. HUMBA participants randomised to placebo capsules received identical looking capsules instead containing microcrystalline cellulose and dextrose anhydrate (Chr. Hansen A/S, Hoersholm, Denmark). The probiotic and placebo capsules were packaged in identical canisters which had been pre-labelled with the participants’ study ID numbers by AnQual Laboratories (School of Pharmacy, University of Auckland), using a password protected pre-assigned random list to ensure participants and the HUMBA researchers were blinded to the intervention allocation. Participants were administered a canister of capsules monthly by a community health worker or a research midwife, and instructed to take one capsule per day.

### Procedure overview

The HUMBA research team visited participants at pre-specified intervals over the course of pregnancy to collect outcome data and administer the capsules. The first (i.e. baseline) assessment visit occurred when the participants were between 12^0^−17 weeks and 6 days pregnant. Eligibility was confirmed, informed consent obtained, and randomisation was conducted. Comprehensive health, sociodemographic, and lifestyle information was obtained, and questionnaires assessing maternal mental health outcomes (depression^40^, anxiety^41^, and functional health and well-being^42^) were administered to participants. The intervention commenced at this visit.

Another assessment visit took place when the participants were 36 weeks pregnant. Questionnaires assessing maternal mental health outcomes were re-administered, and adherence to the intervention (i.e. probiotic/placebo capsule consumption) was assessed.

Additional assessment visits were conducted as part of the wider HUMBA trial, and these are outlined in earlier publications^36,37^.

### Primary outcomes

#### Depression

Depression was assessed using the EPDS^40^, a 10-item self-report scale widely used to screen for perinatal depression by assessing how depressed one has felt over the past 7 days. Although originally developed to screen for postnatal depression, the EPDS is now commonly used to identify depressive symptoms that occur throughout the entire perinatal period. Scale items (e.g. “I have been so unhappy that I have been crying”) are rated on a 4-point Likert scale (0 = “*No, never*”, 3 = “*Yes, most of the time*”). Possible total scores thus range from 0–30, with a cut-off score of ≥13 typically used to indicate probable perinatal depression^34,43^. Scores can also be interpreted continuously, with higher total scores indicating higher levels of depression. The EPDS has been used and validated within samples of pregnant and postnatal New Zealand women, and has been found to have good internal consistency with a Cronbach’s alpha value of 0.86^43,44^. In line with previous studies, the EPDS demonstrated good internal consistency in the current study (α = 0.84).

#### Anxiety

Anxiety was assessed using the 6-item short-form State-Trait Anxiety Inventory (STAI-6)^41^. The STAI-6 is a shortened version of the original Spielberger State-Trait Anxiety Inventory (STAI)^45^, and is a commonly used anxiety screening tool which specifically assesses state anxiety (i.e. how anxious a person is feeling right now). The scale items (e.g. “I feel calm”) are rated on a 4-point Likert scale (1 = “*Not at all*”, and 4 = “*Very much*”). Summed scores from all items thus range from 6–24. The summed score is then divided by six and multiplied by twenty in order to generate a new total score within the range of 20–80, which is comparable with the original STAI value range. Different cut-off scores for the STAI (from 39–50) have been used to indicate probable clinically significant anxiety levels in non-pregnant and pregnant samples, with ultimately higher scores indicating greater anxiety^34,46,47^. The STAI-6 has been demonstrated to have good reliability and validity as a screening tool for anxiety during the perinatal period, with Cronbach’s alphas ranging between 0.79 and 0.84^41,48,49^. In the current study the STAI-6 was found to be approaching acceptable internal reliability (α = 0.68).

#### Functional health and well-being

Functional health and well-being was assessed using the 12-Item Short-Form Health Survey (SF-12-v2)^42^ a brief health-related quality of life measure derived from the 36-Item Short-Form Health Survey (SF-36)^50^. The SF-12v2 assesses both physical and mental aspects of functional health and well-being and encompasses eight specific domains (physical functioning, role limitation due to physical functioning, bodily pain, general health perceptions, vitality, social functioning, role limitation due to emotional functioning, and mental health). An example item from the mental health domain is “How much of the time during the past 4 weeks have you felt downhearted and depressed?”, and an example item from the physical functioning domain is “Does your health now limit you in climbing several flights of stairs, and if so, how much?” The anchors used vary depending on each item. It is recommended that the eight domains of the SF-12v2 be aggregated to form two higher-order summary subscales, the Physical Component Summary (PCS) and the Mental Component Summary (MCS), which measure physical and mental functional health and well-being respectively. Higher scores indicate better functional health and well-being. The current study utilised means, standard deviations, and scoring coefficients derived from the New Zealand population to derive the PCS and MCS scores^51^.The SF-12v2 has been found to be valid, reliable, and sensitive to change among diverse samples, including among people with physical health conditions, those undergoing an intervention, and among women during the perinatal period^52^. In the current study, the SF-12v2 demonstrated good internal reliability with a Cronbach’s alpha of 0.82.

### Additional outcomes

#### Adherence

Adherence to the probiotic/placebo intervention was assessed by self-report at 28 weeks and 36 weeks of pregnancy, and post-birth. Participants were asked if they had taken their HUMBA capsule each day, and if not, how many capsules they had missed. An overall binary measure of adherence was created based on the information provided by participants across these time points. Women considered to have taken their capsules at least 75% of the time were classified as adherent.

### Data analysis

Data were collated and analysed using IBM SPSS Statistics Version 25. For all analyses a significance level of *p* < 0.05 was used. Data were screened for errors, and the statistical assumptions for each test were checked. Normality was assessed for continuous outcome variables, with a judgement made based on a combination of visual assessment of histograms, skewness and kurtosis values, and Kolmogrov-Smirnov tests. For outcome variables found not to be normally distributed (depression, anxiety, and MCS scores), log, square root, and reciprocal transformations were trialled. However, these transformations failed to improve the distributions, and thus the variables were left untransformed.

Independent samples t-tests and a series of 2 (group) × 2 (time) mixed analysis of variance tests (ANOVAs) were used to assess the differences in mental health outcomes (depression, anxiety, and PCS scores) between participants allocated to the probiotic or placebo groups over time, testing the hypotheses that women allocated to the probiotic intervention would demonstrate improvements in mental health outcomes from baseline to 36 weeks of pregnancy, as well as whether there was a between-group difference at 36 weeks. An analysis of covariance test (ANCOVA) was used instead of a mixed ANOVA to analyse the MCS scores, as there was a difference in scores between the groups identified at baseline. A sub-group analysis was also performed whereby the ANOVA and ANCOVA analyses were repeated in those participants considered adherent (*n* = 147), in order to assess whether adherence to capsules influenced the results.

While independent samples t-tests and ANOVAs are considered relatively robust to violations of the normality assumption, additional non-parametric tests (Mann-Whitney U and Wilcoxon signed-rank tests) were conducted where required to strengthen confidence in findings. The results of the non-parametric tests were consistent with those produced by the ANOVA analyses and are reported as supplementary information.

## Results

### Baseline demographic and clinical characteristics

As presented in Table 1, the demographic and clinical characteristics of the sample at baseline were largely comparable between the probiotic and placebo groups. Overall, participants were aged between 17 and 45 years (*M* = 29.75, *SD* = 5.45), were ethnically diverse and representative of the CMH region, and the majority were currently employed (55%) and either married or in a civil union (57%). Education levels were mixed. A considerable proportion of the sample was identified as living in areas that fall within the highest deprivation quintile (63%) according to the NZDep2013 index of Deprivation^53^, and all were obese as per the study’s eligibility criteria with BMI values ranging from 30.1 to 62.5 (*M* = 38.69, *SD* = 6.21). Of particular note, 15% of the sample self-reported as having a history of depression, with 2% of the sample being on antidepressant medication at baseline. A slight discrepancy in the number of participants with a history of depression allocated to each group was observed, with 21% of the placebo group and only 9% of the probiotic group having a history of depression. No difference in current antidepressant use was observed.

**Table 1:** Sample Characteristics at Baseline. Legend: ^a^Percentages do not add to 100 due to rounding, ^b^Only applies if participant has had previous pregnancy (*n* = 112).

Variable	Placebo	Probiotic	Total sample
	*n* = 76	*n* = 88	*N* = 164
**Age in years** *M*(*SD*)	29.39 (5.39)	30.06 (5.51)	29.75 (5.45)
**Ethnicity**^a^			
Māori	15 (20%)	18 (21%)	33 (20%)
Pasifika	36 (47%)	42 (48%)	78 (48%)
Asian	5 (7%)	9 (10%)	14 (9%)
Latin American/African	3 (4%)	1 (1%)	4 (2%)
European	17 (22%)	18 (21%)	35 (21%)
**Employment status**			
Employed	38 (50%)	52 (59%)	90 (55%)
Unemployed	38 (50%)	36 (41%)	74 (45%)
**Marital status**^a^			
Married/Civil Union	37 (49%)	56 (64%)	93 (57%)
De facto	31 (41%)	25 (28%)	56 (34%)
Single/Separated	8 (11%)	7 (8%)	15 (9%)
**Highest level of education**			
Did not complete high school	23 (30%)	23 (26%)	46 (28%)
Completed high school	10 (13%)	15 (17%)	25 (15%)
Tertiary education	28 (37%)	32 (36%)	60 (37%)
Other qualification (e.g. diploma)	15 (20%)	18 (21%)	33 (20%)
**Highest deprivation quintile**			
Yes	44 (58%)	59 (67%)	103 (63%)
**BMI** *M*(*SD*)	38.67 (5.97)	38.70 (6.45)	38.69 (6.21)
**First pregnancy**			
Yes	27 (33%)	27 (31%)	52 (32%)
**Pregnancy planned**			
Yes	28 (37%)	39 (44%)	67 (41%)
**Prior pregnancy complication**^b^			
Yes	5 (10%)	8 (13%)	13 (12%)
**History of depression**			
Yes	16 (21%)	8 (9%)	24 (15%)
**Currently using antidepressants**			
Yes	2 (3%)	2 (2%)	4 (2%)
**Currently using antibiotics**			
Yes	7 (9%)	7 (8%)	14 (9%)
**Currently smoking**			
Yes	8 (11%)	8 (9%)	16 (10%)

### Representativeness of the analytic sample

While the analytic sample and those excluded from analyses (i.e. due to the absence of primary outcome data) were comparable on the majority of demographic and clinical characteristics, there were a few exceptions (see Table 2). There was a reduction in mean age for those excluded (*M* = 26.33, *SD* = 5.45) compared to the analytic sample (*M* = 29.75, *SD* = 5.45). There was also a higher rate of single participants (21%) and current smokers (24%) excluded from analyses compared to that of the analytic sample (9% and 10% respectively). The analytic sample and those excluded from analyses did not differ in terms of placebo/probiotic group allocation, nor in terms of mental health outcomes at baseline.

**Table 2:** Comparison of the Analytic Sample and Participants Excluded from Analyses. Legend: ^a^Percentages do not add to 100 due to rounding, ^b^Only applies if participant has had previous pregnancy (*n* = 156), ^c^Missing data for 1 participant (Total *N* = 229, Analytic sample *n* = 164, Exclusions *n* = 65), ^d^Missing data for 3 participants (Total *N* = 227, Analytic sample *n* = 162, Exclusions *n* = 64.

Variable	Analytic Sample	Exclusions
	*n* = 164	*n* = 66
**Age in years** *M*(*SD*)	29.75 (5.45)	26.33 (5.45)
**Ethnicity**^a^		
Māori	33 (20%)	19 (29%)
Pasifika	78 (48%)	36 (55%)
Asian	14 (9%)	4 (6%)
Latin American/African	4 (2%)	0 (0%)
European	35 (21%)	7 (11%)
**Employment status**		
Employed	90 (55%)	27 (41%)
Unemployed	74 (45%)	3 (59%)
**Marital status**^a^		
Married/Civil Union	93 (57%)	31 (47%)
De facto	56 (34%)	21 (32%)
Single/Separated	15 (9%)	14 (21%)
**Highest level of education**		
Did not complete high school	46 (28%)	23 (35%)
Completed high school	25 (15%)	4 (6%)
Tertiary education	60 (37%)	23 (35%)
Other qualification (e.g. diploma)	33 (20%)	16 (24%)
**Highest deprivation quintile**		
Yes	103 (63%)	45 (68%)
**BMI** *M*(*SD*)	38.69 (6.21)	38.23 (5.78)
**First pregnancy**		
Yes	52 (32%)	22 (33%)
**Pregnancy planned**		
Yes	67 (41%)	29 (44%)
**Prior pregnancy complication**^b^		
Yes	13 (12%)	9 (21%)
**History of depression**		
Yes	24 (15%)	6 (9%)
**Currently using antidepressants**^a^		
Yes	4 (2%)	3 (5%)
**Currently using antibiotics**		
Yes	14 (9%)	8 (12%)
**Currently smoking**		
Yes	16 (10%)	16 (24%)
**Depression at baseline**^c^		
Score *M*(*SD*)	7.23 (4.61)	7.52 (5.26)
Yes (Score ≥ 13)	22 (13%)	9 (14%)
**Anxiety score at baseline**^c^	27.83 (8.87)	28.36 (10.33)
**Functional health and well-being score at baseline**^d^		
PCS	42.57 (9.04)	41.74 (8.35)
MCS	46.27 (9.41)	46.75 (10.22)
**Group allocation**		
Placebo	76 (46%)	39 (59%)
Probiotic	88 (54%)	27 (41%)

### Mental health outcomes

Mental health outcomes at baseline and at follow-up (36 weeks of pregnancy) are displayed in Table 3. At baseline, the mental health outcomes of the probiotic and placebo groups were comparable, with the exception of MCS scores which were slightly higher in the probiotic group (*M* = 48.02, *SD* = 8.49) compared to the placebo group (*M* = 44.30, *SD* = 10.05).

**Table 3:** Mental Health Outcomes at Baseline and 36 Weeks of Pregnancy. Legend: ^a^Percentages do not add to 100 due to rounding, bMissing data for 5 participants (Total *N* = 159, Placebo *n* = 73, Probiotic *n* = 86)^, c^Missing data for 2 participants (Total *N* = 162, Placebo *n* = 76, Probiotic *n* = 86), ^d^Missing data for 8 participants (Total *N* = 156, Placebo *n* = 74, Probiotic *n* = 82).

Variable		Placebo	Probiotic	Mean difference	*p* value	Total sample
		*n* = 76	*n* = 88	[95% CI]		*N* = 164
**Depression**						
**Baseline**	Score *M* (*SD*)	7.49 (4.98)	7.00 (4.28)	−0.49 [−0.94, 1.92]		7.23 (4.61)
	Yes (score ≥ 13)	12 (16%)	10 (11%)			22 (13%)
	No (score ≤ 12)	64 (84%)	78 (89%)			142 (87%)
**36 weeks**^a^	Score *M* (*SD*)	6.76 (4.65)	7.18 (3.80)	0.42 [−1.72, 0.89]	0.527	6.99 (4.21)
	Yes (score ≥ 13)	8 (11%)	8 (9%)			16 (10%)
	No (score ≤ 12)	68 (90%)	80 (91%)			148 (90%)
**Change score**	*M*(*SD*)	−0.72 (5.11)	0.18 (4.09)	−0.91 [−2.35, 0.54]]	0.217	−0.24 (4.60)
**Anxiety**						
**Baseline**	Score *M*(*SD*)	27.72 (9.88)	27.92 (7.95)	0.20 [−2.95, 2.55]		27.83 (8.87)
**36 weeks**^b^	Score *M*(*SD*)	32.88 (10.31)	31.94 (10.22)	−0.94 [−2.29, 4.17]	0.566	32.37 (10.24)
**Change score**^b^	*M*(*SD*)	5.34 (11.02)	3.92 (11.13)	1.43 [−2.06, 4.91]]	0.419	4.57 (11.07)
**Functional health and well-being**						
**Baseline**^c^	Score *M*(*SD*)					
	PCS	41.34 (9.00)	43.65 (8.99)	2.30 [−5.10, 0.50]		42.57 (9.04)
	MCS	44.30 (10.05)	48.02 (8.49)	3.72 [−6.59, −0.84]		46.27 (9.41)
**36 weeks**^d^	Score *M*(*SD*)					
	PCS	35.90 (8.63)	36.77 (9.75)	0.88 [−3.72, 2.02]	0.801	36.36 (9.21)
	MCS	48.31 (9.89)	48.62 (8.56)	0.38 [−3.32, 2.57]	0.550	48.47 (9.19)
**Change score**^d^	*M*(*SD*)					
	PCS	−5.40 (11.59)	−6.93 (9.06)	1.53 [−1.74, 4.81]	0.357	−6.20 (10.33)
	MCS	4.23 (10.33)	0.71 (10.32)	3.52 [0.25, 6.79]	0.035	2.38 (10.44)

In terms of the follow-up data, independent samples t-tests revealed that mental health outcomes did not significantly differ between the probiotic and placebo groups at 36 weeks of pregnancy. These mental health outcomes in relation to the effects of the intervention are discussed further in the primary analyses section below. As noted, comparable results produced using non-parametric tests are viewable as supplementary information.

### Primary analyses: effect of the probiotic intervention on mental health outcomes

#### Depression

Analysis of depression scores revealed no significant main effect of time, *F*(1, 162) = 0.57, *p* = 0.452, ƞ_p_^2^ = 0.00, indicating that irrespective of group allocation, there was no change in depression scores over time. The main effect of group allocation was also non-significant, *F*(1, 162) = 0.00, *p* = 0.954, ƞ_p_^2^ = 0.00, indicating that irrespective of time point, there was no difference in depression scores between women allocated to the probiotic or placebo group. There was also no significant interaction effect observed between time and group allocation, *F*(1, 162) = 1.59, *p* = 0.209, ƞ_p_^2^ = 0.01.

#### Anxiety

Analysis of anxiety scores revealed a significant main effect of time, *F*(1, 157) = 27.54, *p* < 0.001, ƞ_p_^2^ = 0.15, indicating that anxiety scores increased between baseline (*M* = 27.80, *SD* = 8.94) and 36 weeks of pregnancy (*M* = 32.37, *SD* = 10.24), irrespective of group allocation. There was no significant main effect of group allocation, *F*(1, 157) = 0.03, *p* = 0.858, ƞ_p_^2^ = 0.00, indicating that there was no difference in anxiety scores between women allocated to the probiotic or placebo group, irrespective of time point. There was no significant interaction effect observed between time and group allocation, *F*(1, 157) = 0.66, *p* = 0.209, ƞ_p_^2^ = 0.00.

#### Functional health and well-being – Physical

Analysis of PCS scores revealed a significant main effect of time, *F*(1, 154) = 55.25, *p* < 0.001, ƞ_p_^2^ = 0.26. indicating that physical health and well-being scores decreased between baseline (*M* = 42.56, *SD* = 9.07) and 36 weeks of pregnancy (*M* = 36.36, *SD* = 9.21), irrespective of group allocation. There was no significant main effect of group allocation, *F*(1, 154) = 1.83, *p* = 0.179, ƞ_p_^2^ = 0.01, indicating that there was no difference in physical health and well-being scores between women allocated to the probiotic or placebo group, irrespective of time point. There was also no significant interaction effect observed between time and group allocation, *F*(1, 154) = 0.85, *p* = 0.357, ƞ_p_^2^ = 0.01.

#### Functional health and well-being – Mental

Analysis of MCS scores revealed that after adjusting for baseline scores, there was no significant difference in MCS scores at 36 weeks of pregnancy between the probiotic and placebo groups, *F*(1, 153) = 0.66, *p* = 0 0.419, ƞ_p_^2^ = 0.00. Without adjusting for baseline scores, there is a significant difference in MCS scores (*p* = 0.035) between groups over time, with the placebo group demonstrating higher scores (see Table 3).

#### Adherence

As discussed, primary analyses assessing the effect of the probiotic intervention on mental health outcomes were replicated including only those participants considered adherent (n = 147). The significance of main effects and interaction effects did not differ from those of the original analyses including all participants. See supplementary information for replicated ANOVA and ANCOVA results.

## Discussion

The purpose of this study was to investigate the influence of probiotic capsules on mental health outcomes among a multi-ethnic and high-deprivation sample of pregnant women with obesity in New Zealand. Through this pre-specified secondary analysis of the HUMBA trial we found that the probiotic capsules containing *Lactobacillus rhamnosus* GG and *Bifidobacterium lactis* BB12 did not improve depression, anxiety, or functional health and well-being scores from baseline to 36 weeks of pregnancy, or demonstrate a between-group difference at 36 weeks. These findings were in contrast to our hypotheses. Depression scores remained stable across the two time points, whereas anxiety scores increased, and physical well-being (PCS) scores decreased, irrespective of group allocation. Mental well-being (MCS) scores did not differ between the two groups at 36 weeks of pregnancy.

Research regarding probiotics and mental health is at an emergent stage, with results from heterogeneous human randomised controlled trials remaining equivocal^54–56^. Our study findings are in contrast to the single previous study that investigated the effect of probiotics in pregnancy on mental health outcomes. Slykerman *et al*.^34^, found that pregnant women allocated to receive the probiotic *Lactobacillus rhamnosus* HN001 reported significantly lower anxiety and depression scores in the postnatal period compared to those who received a placebo. However these results need to be interpreted with caution as they relied on retrospective self-reported mental health information (i.e. mothers were approached at 6 or 12 months post-birth and asked to think back to how they felt when their child was 1–2 months old), and thus there is potential for measurement error. Additionally, between 80–90% of the pregnant women included in the study had a history of asthma, eczema, or hayfever requiring medication, and overall the sample was non-deprived, non-obese, highly educated, and predominantly European. The results of this study may therefore not be generalisable to other populations, and these demographic differences may in part explain the conflicting results with our study. The results of Slykerman *et al*.’s study require replication.

In addition to the methodological limitation and demographic differences between our study and Slykerman *et al*.’s outlined above, the contrasting results could be attributable to the different strains of probiotic utilised. Research indicates that effects of probiotics are strain specific, and while most studies investigating probiotics and mental health outcomes have utilised strains from the *Bifidobacterium* or *Lactobacillus* genera of bacteria (as in the case of our study and Slykerman *et al.’s*), there is significant heterogeneity in the exact strains used^56–59^. This heterogeneity has so far prevented any consensus being formed regarding which strains are most efficacious and for whom^54,55,57,60^. It is possible that the probiotic strains employed in the current study (*Lactobacillus rhamnosus* GG and *Bifidobacterium lactis* BB12) were not optimal in terms of improving mental health outcomes in the sample. This probiotic formulation was used as it had been safely and efficaciously used previously within a sample of pregnant women in a Finnish randomised controlled trial^32^, with results revealing positive metabolic outcomes. More research investigating mental health outcomes in relation to the use of *Lactobacillus rhamnosus* GG and *Bifidobacterium lactis* BB12 is required before the psychobiotic effects of this formulation can be substantiated or discounted.

In addition to the lack of consensus regarding optimal probiotic strains, there is no consensus regarding the dosage of probiotics required to produce clinical effects^54,59,61,62^. The dosage employed in the current study was 6.5 × 10^9^ CFU, and it remains unclear whether this concentration was adequate or optimal to influence mental health within the current sample. Previous studies such as that by Whorwell *et al*.^63^, have demonstrated that different dosages of the same probiotic can produce meaningfully different effects. Whorwell *et al*.^63^ found that only one of three specific dosages of *Bifidobacterium infantis* 35624 administered (1 × 10^8^ CFU/mL) was effective at reducing abdominal pain and other Irritable Bowel Syndrome (IBS) related symptoms, including quality of life, in a sample of women with IBS. One of the other doses (10^10^ CFU/mL) was associated with significant formulation problems resulting in it being unable to proliferate in the gastrointestinal tract and thus unable to exert positive effects. Further investigation into the required dosage of probiotic formulations is thus necessary.

Further comparison of our study’s results with those from studies exploring probiotics and mental health in non-pregnant and non-clinical populations (i.e. not depressed or anxious) provide some interesting insights. For example, studies by Benton *et al*.^64^, and Steenbergen *et al*.^65^ also did not find improvements in self-reported mental health as a result of probiotic consumption. Low levels of psychopathology present in samples at baseline has been suggested as a potential factor contributing to these results^54,64,65^. This assertion is supported by the study of Benton *et al*.^64^, as when they conducted further analyses with participants whose mood was initially poor, improvements in self-reported mood associated with probiotic consumption were observed. A recent meta-analysis on the use of probiotics to alleviate depressive symptoms provides further support for this notion, as while the overall effect of probiotics on mood was found to be non-significant, a statistically significant benefit was observed in a subgroup analysis including only studies conducted in depressed samples, after studies conducted in ‘healthy’ individuals were excluded^54^. Given that depression and anxiety symptoms in the current study were relatively low at baseline (i.e. mean EPDS score of 7.23/30, and mean STAI-6 score of 27.83/80) thereby creating a floor effect, this may have made it difficult to detect an improvement, and thus in part explain the results.

However, while the baseline level of psychopathology in the sample may explain why no change in depression scores was observed, this argument does not explain why anxiety rates increased from baseline to 36 weeks of pregnancy, nor why physical well-being scores decreased. Our findings are consistent with previous non-intervention based pregnancy research, in which anxiety has been shown to increase, and physical well-being to decrease, as pregnancy progresses^19,66–68^. While it was hypothesised that the probiotic intervention would attenuate or overcome these trajectories, this was not supported by the results of the current study.

In addition to restricted antenatal follow-up of the current study, a further limitation is the lack of an objective measure of capsule consumption. Adherence to capsule consumption was measured via self-report, and it is well-recognised that self-reported adherence is prone to biases such as socially desirable responding and recall bias^69,70^. While efforts were made to reduce these biases, it would have been beneficial (although it was not feasible) to corroborate this self-reported information with a further alternative measure of adherence such as pill counts or an electronic pill bottle monitoring system. Capsule consumption could also have been objectively measured by obtaining faecal samples from participants and assessing them for presence of probiotic bacteria. This method would have provided not only more insight into adherence of the intervention, but also demonstrated whether or not the probiotic formulation utilised was able to survive transit in the gastrointestinal tract and remain viable^58,61,62^. While this approach was attempted, few women consented to the collection of stool samples.

Another limitation of this study relates to the restricted sample size. While a total of 230 women were recruited, primary outcome data pertaining to the current study (depression scores at 36 weeks of pregnancy) were only able to be obtained for 164 women (i.e. 71%). As noted in the results, there were some differences between those excluded from analyses and those included within the analytic sample. Specifically, there were more younger, single, and currently smoking participants excluded from analyses. Those excluded were also further along in pregnancy, likely reflecting a difficulty in arranging the initial baseline assessment visit, and thus possibly the presence of more challenging life circumstances. All of these differences may have impacted on the results of the study, given that young maternal age, being single, smoking, and having challenging life circumstances have been associated with poorer maternal mental health outcomes^2^. Additionally, the generalisability of the findings may be affected, as these characteristics are underrepresented in the analytic sample. However, the analytic sample and those excluded from analyses did not differ in terms of placebo/probiotic group allocation, nor in terms of mental health outcomes at baseline, and this arguably attenuates the potentially negative impacts of these differences and suggests that the results are valid. The power calculation was also conducted retrospectively using the sample of *n* = 164, ensuring ability to detect a meaningful effect.

On the basis of the results from this study, the use of probiotics for mental health benefits cannot be recommended for pregnant women with obesity. However, as this is the first randomised controlled trial to have explored the influence of probiotics on mental health outcomes in a sample of pregnant women with obesity, more studies are warranted. The findings of this study further contribute to the complexity of knowledge surrounding the gut-brain axis as a theoretical framework, and highlight remaining ‘unknowns’ pertaining to the gut-brain axis. The role of the gut-brain axis in the specific contexts of obesity and pregnancy remains unclear, and thus, whether or not probiotics are a useful or appropriate means by which to intervene and improve outcomes remains to be determined.

In conclusion, this study has extended current understandings regarding the use of probiotics for mental health and has addressed a significant gap in the literature by exploring this concept within the context of a high deprivation, multi-ethnic New Zealand sample of pregnant women with obesity. While no beneficial effects of probiotics on mental health outcomes were observed, further studies are required to validate and build on these findings. Probiotics represent an emergent area of research, and many questions regarding their use remain.

## Supplementary information

Supplementary Information.

## Data Availability

All applications for additional data sharing will be considered by the HUMBA Trial Steering Committee.
