# Supplementary material for: Probiotics and Maternal Mental Health: A Randomised Controlled Trial among Pregnant Women with Obesity
Source: Sci Rep. 2020 Jan 28;10:1291. doi: 10.1038/s41598-020-58129-w (PMC6987087; doi:10.1038/s41598-020-58129-w)
Supplement: Supplementary file 1 — Supplementary Information. [file 41598_2020_58129_MOESM1_ESM.docx]

**Supplementary Information**

**Probiotics and Maternal Mental Health: A Randomised Controlled Trial among Pregnant Women with Obesity**

**Julia P. Dawe^1^, Lesley M. E. McCowan^2^, Jess Wilson^2^, Karaponi A. M. Okesene-Gafa^2^, & Anna S. Serlachius^1,*^**

^1^Department of Psychological Medicine, Faculty of Medical and Health Sciences, The University of Auckland, 1142, Auckland, New Zealand

^2^Department of Obstetrics and Gynaecology, Faculty of Medical and Health Sciences, The University of Auckland, 1142, Auckland, New Zealand

*a.serlachius@auckland.ac.nz

**Non-Parametric tests replicating primary analyses assessing effect of the probiotic intervention on mental health outcomes**

Given minor violations of the normality assumption in the depression, anxiety, and MCS score data, Wilcoxon Signed Rank tests and Mann-Whitney U tests were conducted in order to provide confidence in the results obtained from the mixed ANOVA and ANCOVA analyses.

**Depression.** In line with the findings from the mixed ANOVA, results from the Wilcoxon Signed Rank test revealed no significant change in depression scores between baseline (*Mdn* = 7.00) and 36 weeks of pregnancy (*Mdn* = 7.00) for the probiotic group, *z* = -1.18, *p* = 0.238, *r =* 0.09. Results from the Mann-Whitney U test also found no difference in depression scores at 36 weeks of pregnancy between participants allocated to the probiotic group (*Mdn* = 7.00, *n* = 88) or placebo group (*Mdn* = 6.00, *n* = 76), *U* = 3074, *z* = -0.89, *p =* 0.372, *r* = 0.07.

**Anxiety.** In line with the findings from the mixed ANOVA, results from the Wilcoxon Signed Rank tests revealed a significant increase in anxiety scores between baseline (*Mdn* = 26.67) and 36 weeks of pregnancy (*Mdn* = 30.00) for the probiotic group, *z* = -3.14, *p* = 0.002., *r =* 0.24, and similarly, a significant increase in anxiety scores between baseline (*Mdn* = 23.33) and 36 weeks of pregnancy (*Mdn* = 30.00) for the placebo group, *z* = -3.74, *p* < 0.001, *r =* 0.31. Results from the Mann-Whitney U test also suggest there was no difference in anxiety scores at 36 weeks of pregnancy between participants allocated to the probiotic group (*Mdn* = 30.00, *n* = 86) or placebo group (*Mdn* = 30.00, *n* = 73), *U* = 2962, *z* = -0.62, *p =* 0.538, *r* = 0.05.

**Functional health and well-being – Mental.** In line with the findings from the ANCOVA, results from the Mann-Whitney U test suggest there was no difference in mental well-being scores at 36 weeks of pregnancy between participants allocated to the probiotic group (*Mdn* = 50.01 , *n* = 82) or placebo group (*Mdn* = 50.73 , *n* = 74), *U* = 3021.50, *z* = -.04, *p =* 0.965 , *r* = 0.00.

**Adherence Analyses: Replication of primary analyses assessing effect of the probiotic intervention on mental health outcomes with only participants considered adherent (n=147)**

***Depression.*** The results of the mixed ANOVA with depression scores revealed no significant main effect of time (*F*(1, 145) = 0.00, *p* = 0.962, ƞp2 = 0.00), no significant main effect of group allocation (*F*(1, 145) = 0.03, *p* = 0.869, ƞp2 = 0.00), and no significant interaction effect (*F*(1, 145) = 3.23, *p* = 0.074, ƞp2 = 0.02).

***Anxiety.*** The results of the mixed ANOVA with anxiety scores revealed a significant main effect of time (*F*(1, 140) = 32.35, *p* < 0.001, ƞp2 = 0.19), no significant main effect of group allocation (*F*(1, 140) = 0.09, *p* = 0.760, ƞp2 = 0.00), and no significant interaction effect (*F*(1, 140) = 0.53, *p* = 0.468, ƞp2 = 0.00).

***Functional health and well-being – Physical.*** The results of the mixed ANOVA with PCS scores revealed a significant main effect of time (*F*(1, 139 = 54.07, *p* < 0.001, ƞp2 = 0.28), no significant main effect of group allocation (*F*(1, 139) = 2.83, *p* = 0.095, ƞp2 = 0.02), and no significant interaction effect (*F*(1, 139) = 1.93, *p* = 0.167, ƞp2 = 0.01),

***Functional health and well-being – Mental.*** The ANCOVA with MCS scores showed that after adjusting for baseline scores, there was no significant difference in MCS scores at 36 weeks of pregnancy between the probiotic and placebo group, *F*(1, 138) = 0.70, *p* = 0.404, ƞp2 = 0.01.
